# Examining a conceptual model of parental nurturance, parenting practices and physical activity among 5–6 year olds

**DOI:** 10.1016/j.socscimed.2015.11.022

**Published:** 2016-01

**Authors:** Simon J. Sebire, Russell Jago, Lesley Wood, Janice L. Thompson, Jezmond Zahra, Deborah A. Lawlor

**Affiliations:** a Centre for Exercise, Nutrition and Health Sciences, School for Policy Studies, University of Bristol, United Kingdom; b School of Sport, Exercise and Rehabilitation Sciences, University of Birmingham, United Kingdom; c MRC Integrative Epidemiology Unit at the University of Bristol and School of Social and Community Medicine, University of Bristol, United Kingdom

**Keywords:** Physical activity, Parenting, Children, Mediation

## Abstract

**Rationale:**

Parenting is an often-studied correlate of children's physical activity, however there is little research examining the associations between parenting styles, practices and the physical activity of younger children.

**Objective:**

This study aimed to investigate whether physical activity-based parenting practices mediate the association between parenting styles and 5–6 year-old children's objectively-assessed physical activity.

**Methods:**

770 parents self-reported parenting style (nurturance and control) and physical activity-based parenting practices (logistic and modeling support). Their 5–6 year old child wore an accelerometer for five days to measure moderate-to-vigorous physical activity (MVPA). Linear regression was used to examine direct and indirect (mediation) associations. Data were collected in the United Kingdom in 2012/13 and analyzed in 2014.

**Results:**

Parent nurturance was positively associated with provision of modeling (adjusted unstandardized coefficient, β = 0.11; 95% *CI* = 0.02, 0.21) and logistic support (β = 0.14; 0.07, 0.21). Modeling support was associated with greater child MVPA (β = 2.41; 0.23, 4.60) and a small indirect path from parent nurturance to child's MVPA was identified (β = 0.27; 0.04, 0.70).

**Conclusions:**

Physical activity-based parenting practices are more strongly associated with 5–6 year old children's MVPA than parenting styles. Further research examining conceptual models of parenting is needed to understand in more depth the possible antecedents to adaptive parenting practices beyond parenting styles.

## Highlights

• A conceptual model of parenting and children's physical activity was tested.
• Physical activity was measured objectively using accelerometers.
• Parenting practices were associated with greater physical activity of 5–6 year olds.
• Nurturing parenting was positively associated with physical activity parenting.

## 1 Introduction

Establishing physical activity in early childhood is important because it is associated with physical, psychological and social benefits (Janssen and LeBlanc, 2010). In the United Kingdom (UK), 5–18 year olds are recommended to perform moderate-to-vigorous intensity physical activity (MVPA) for at least 60 min per day (Department of Health (2011)) with comparable guidelines in the United States (US) (Physical Activity Guidelines Advisory Committee, 2008). The Health Survey for England (Craig and Mindell, 2013) indicates that 79% of boys and 84% girls aged 5–15 years in the UK do not meet this recommendation. Levels of physical activity are similar among youth aged 12 to 15 in the US (73% of boys and 78% girls not meeting recommendations) (Fakhouri et al., 2014). As physical activity is estimated to decrease by 7% per year during adolescence (Dumith et al., 2011), it is important to foster health-promoting levels of physical activity in childhood that can be maintained in later life.

Parents are hypothesized to play a central role in their children's physical activity (Patrick et al., 2013). This influence can include general parenting styles and specific parenting practices. General parenting styles refer to *how* parents interact with their children and the “*emotional and relational climate*” that they create (Patrick et al., 2013, p. S74). Parenting styles are characterized by dimensions of: (a) responsiveness/nurturance, which refers to a parent's efforts to encourage individuality and self-regulation by responding to the child's needs, offering emotional support and being involved; and (b) demandingness/control, which reflects the extent to which parents make demands such as setting rules, and guide behavior through restrictive and punitive means (Baumrind, 1971, Baumrind, 1991, Maccoby and Martin, 1983). Evidence for an association between parenting styles and children's physical activity is mixed (Sleddens et al., 2011). The most consistent findings in a recent systematic review (Sleddens et al., 2011) were: (a) that parent control was not associated with young people's physical activity, and (b) that physical activity was more often positively associated with positive parenting approaches, such as an authoritative parenting style and parental nurturance, in both cross-sectional and longitudinal studies.

Physical activity parenting practices refer to *what* parents do; that is, the specific actions undertaken to encourage their child to be physically active (Patrick et al., 2013) including providing transport, funding, or equipment (logistic support) and by being active themselves (modeling support) (Davison and Jago, 2009, Edwardson and Gorely, 2010). A recent review of systematic reviews reported an overall positive association between physical activity parenting practices (e.g., parental encouragement, provision of transportation and involvement) and young people's physical activity (Biddle et al., 2011). However, the majority of studies included focused on adolescents rather than younger children. Early childhood is an important time for the development and refinement of fundamental movement skills which underpin physical activity in later life (Lubans et al., 2010). As such, understanding how parents may facilitate or undermine exposure to physical activity in early childhood is important. A recent meta-analysis of 38 associations from 12 studies (Mitchell et al., 2012) identified a moderate positive association between parental influence (defined as parent physical activity and encouragement of their child) and the physical activity of 2–7 year olds. However, only three of the quantitative studies reviewed included children who were older than pre-school age (Alderman et al., 2010, Davison and Birch, 2001, Moore et al., 1991). All three studies assessed the direct association between parent and child physical activity rather than the association between parenting practices and child activity, and produced inconsistent findings (either positive or null associations). In addition to the narrow range of parent variables assessed, the samples in these studies were small (*n* = 68 to 197) and only one assessed physical activity objectively. Therefore, studies with larger samples and objective measures of activity are needed to examine the associations between physical activity-based parenting practices and children's physical activity (Trost et al., 2013).

**Fig. 1:**
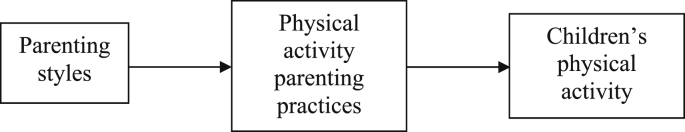
Conceptual model hypothesizing mediation of parenting styles by physical activity parenting practices.

Little research has explored the mechanisms by which parenting styles and practices might influence children's physical activity (Patrick et al., 2013). Recently, conceptual models have been proposed which hypothesize that: (a) parenting styles could moderate the association between parenting practices and physical activity (i.e., parenting styles and practices interact synergistically or antagonistically to affect child activity), and (b) the effect of parenting styles on physical activity could be mediated by parenting practices (i.e., there is a causal chain in which parenting styles lead to parenting practices, which in turn lead to child activity) (Patrick et al., 2013, Sleddens et al., 2011). While two studies (Hennessy et al., 2010, Langer et al., 2014) have identified that parenting styles may interact with or moderate the association between parenting practices and the activity of 5–11 year olds there is no research that has assessed the mediation hypothesis (Patrick et al., 2013, Sleddens et al., 2011) (see Fig. 1). This model proposes that parents' general parenting style is *causally* associated with their use of parenting practices, which are targeted at lifestyle behaviors such as physical activity. A possible scenario in which general parenting styles could be a pre-cursor to behavior-specific parenting practices is a parent who adopts a nurturing parenting approach being more likely to empathically model the behavior that they want to promote for their child (e.g., being physically active themselves). Alternatively, a parent who takes a more controlling parenting approach may be less likely to model the promoted behavior in favor of supporting it with more logistic strategies (e.g., paying for a sports club membership).

The aim of this study was to examine associations between parenting styles and practices and 5–6 year old children's physical activity and whether parenting practices played a mediating role between parenting styles and physical activity.

## 2 Methods

### 2.1 Study design

Data are from a cross-sectional study (B-ProAct1v) which aimed to understand the correlates of physical activity and screen-viewing among 5–6 year old children from 57 schools in Bristol, UK and the surrounding area. Study design, participant recruitment and accelerometer and covariate data collection are described in greater detail elsewhere (Jago et al., 2014). In 2012/13 a parent (mother or father) completed questionnaires assessing parental influences on children's physical activity and screen-viewing including physical activity parenting practices and parenting styles. Children wore an accelerometer to assess physical activity as described below. Ethical approval was given by the School for Policy Studies Ethics Committee at the University of Bristol and parents provided written informed consent for themselves and their child to participate voluntarily.

### 2.2 Measures

#### 2.2.1 Physical activity

Children were asked to wear an ActiGraph GT3X accelerometer (ActiGraph, LLC, Fort Walton Beach, FL) on an elastic belt positioned on their hip for five days including a weekend. The ActiGraph GT3X is a triaxial accelerometer although in this study only acceleration in the vertical plane was analyzed as the cut-points used to determine the intensity of physical activity were derived from vertical acceleration data. Data were recorded in raw form and downloaded in 10 s epochs. Participants who provided at least three valid days (i.e., 500 min of data, after excluding intervals ≥60 min of zero counts allowing up to two minutes of interruptions) were included in analysis. Uniaxial data were analyzed to estimate the minutes spent in MVPA per day using the age-appropriate Evenson threshold (Evenson et al., 2008). These thresholds were developed by directly comparing accelerometer data against an objective measure of energy expenditure (i.e., indirect calorimetry) among the same sample of children while they performed various activities with different intensity levels. In a comparative study with other widely-used accelerometer cut-points, the Evenson thresholds (in which stair climbing and brisk walking corresponded to moderate intensity physical activity) were shown to provide the most accurate assessments of children's energy expenditure (Trost et al., 2011).

#### 2.2.2 Parenting style

The Parenting Dimensions Inventory-Short (PDI-S) (Power, 2002) was used to measure parental nurturance and control. Nurturance (i.e., warmth, involvement, appreciation and respect) was assessed with six items (e.g., *I respect my child's opinion and encourage him/her to express it*) scored on a six-point Likert scale (1 = *Not at all like me* to 6 = *Exactly like me*). A total nurturance score (possible range 0–36) was calculated by summing all items, and an average nurturance score (possible range 0–6) was calculated by dividing the total by six. The average score was used in analysis. The internal consistency of the scale was α = 0.87. Parental control was measured with five items that assessed the degree to which the parent values practices such as obedience, setting and enforcing rules and guiding behavior. Items are presented using a dichotomous choice format in which parents endorsed one of two conflicting statements (e.g., *I care more than most parents I know about having my child obey me* vs. *I care less than most parents I know about having my child obey me)*. Following the reverse scoring of two items, items were summed to provide a control score (possible range 0–5). The internal consistency of the scale was low in the present sample (α = 0.27) and was not improved by item-deletion. As such, parental control was excluded from further analysis.

#### 2.2.3 Parenting practices

Parents completed the logistic support (3 items, e.g., *I take my child places where he/she can be physically active*) and parental modeling (3 items, e.g., *I encourage my child to be physically active by leading by example – by being a role model*) subscales of the Activity Support Scale (Davison et al., 2011). Items were scored on a four-point Likert type scale (1 = *strongly disagree* to 4 = *strongly agree*) and averaged within subscales to provide logistic support (α = 0.73) and modeling (α = 0.86) scores.

#### 2.2.4 Covariates

Parents reported their gender and the age and gender of their participating child. Children's height and weight were measured to the nearest 0.1 cm and 0.1 kg respectively after they removed shoes and outer clothing. Height and weight were used to calculate age and gender-specific standardized child body mass index (BMI) (kg/m^2^) z-score (Standard Deviation Score [SDS]) using UK reference curves (Cole et al., 1995, Vidmar et al., 2004). Level of deprivation was derived using the Index of Multiple Deprivation (IMD) (http://data.gov.uk/dataset/index-of-multiple-deprivation) based on participants' home postcode. Higher scores represent greater deprivation.

### 2.3 Statistical analysis

Only participants with complete data were included in analysis (i.e., participants were excluded if they had missing data for one or more study variable). Missing data were not imputed. From the original sample of 1456 pupils, 1023 (70.3%) children met the accelerometer inclusion criteria, and of these, 900 (88.0%) parents provided complete psychosocial data. Of these cases, 770 parent–child dyads (85.6%) provided complete demographic data and were therefore retained for the present analyses.

The associations between parental nurturance, practices and children's physical activity and the mediation hypothesis of the conceptual model (Patrick et al., 2013, Sleddens et al., 2011) were examined using sequential multivariable linear regression and examination of indirect effects (Cerin and Mackinnon, 2009, MacKinnon, 2007, MacKinnon et al., 2007). Specifically, regression models were used to estimate: (a) the association between parental nurturance and child MVPA; (b) the association between nurturance and each of the proposed parenting practice mediators (i.e., logistic and modeling support with mutual adjustment for each other); (c) the associations between logistic and modeling support and child MVPA, mutually adjusted for each practice and nurturance; and (d) the indirect association between nurturance and child MVPA through the proposed mediators (logistic support and/or modeling). Mediation was assessed by examining bootstrapped, bias-corrected confidence intervals of the indirect effects of nurturance on MVPA via logistic support and modeling separately, requesting 5000 bootstrap replications (Cerin and Mackinnon, 2009, MacKinnon, 2007, MacKinnon et al., 2007). As a supplementary analysis, we stratified the mediation analysis by child gender and subjectively compared the magnitude of association between gender-specific subgroups by examining the point estimates (see Supplemental Table 1). We also tested statistically for evidence of heterogeneity (i.e., difference in the magnitude of association between the subgroups) by including an interaction term for each exposure*child gender. In further supplementary analysis, we used multivariable linear regression analysis to examine whether parent nurturance moderated any association between parenting practices and children's MVPA by including interaction terms for logistic support*nurturance and modeling support*nurturance. Each analysis was adjusted for child gender, parent gender, child z-BMI, and deprivation (Supplemental Table 1). Robust standard errors were used to account for the clustering of children within schools. Unstandardized regression coefficients (β) are presented for all analyses. All analyses were conducted in 2014 using Stata version 12.1 (Statacorp, College Station, TX).

## 3 Results

### 3.1 Sample

The included sample consisted of 404 boys (*M* age = 6.02, *SD* = 0.42), 366 girls (*M* age = 5.98, *SD* = 0.44), 572 mothers (*M* age = 37.4, *SD* = 5.3) and 198 fathers (*M* age = 39.7, *SD* = 5.5). Parents included in the analysis were less deprived (*p* < 0.001), more nurturing (*p* = 0.02) and provided more logistic support (*p* = 0.001) than those excluded due to missing data. Included children had lower BMI (SDS) than those excluded (*p* = 0.005), but engaged in similar levels of physical activity (*p* = 0.33).

On average, children performed approximately 67 min of MVPA per day (Table 1) and 62.6% (*n* = 482) met the UK physical activity guidelines that recommend 60 min MVPA per day on average. Parents reported providing marginally greater modeling than logistic support.

Associations between exposures and outcomes and the mediation results are presented in Table 2. Parental nurturance was not directly associated with child MVPA. Nurturance was associated with the provision of modeling support (β = 0.11, 95% *CI* = 0.02 to 0.21) and logistic support (β = 0.14, 95% *CI* = 0.07 to 0.21). Modeling support was associated with higher child MVPA, specifically, for every one unit increase in parents' modeling support there was a 2.41 min increase in MVPA per day (95% *CI* = 0.23 to 4.60). There was weaker evidence for a positive association between logistic support and MVPA (β = 2.41, 95% *CI* = −0.42 to 5.25). Mediation analysis showed was a small indirect effect of parental nurturance on children's MVPA via parent modeling (β = 0.27, 95% *CI* = 0.04 to 0.70).

In the mediation analysis stratified by child gender, the point estimates suggested that there was a possible inverse association between nurturance and MVPA among girls (β = −2.90, 95% *CI* = −5.84 to 0.04) (i.e., more nurturance being associated with less time spent in MVPA) and a null association amongst boys (Supplemental Table 1). However, there was no statistical evidence for a difference in this association between boys and girls (*P* for Heterogeneity = 0.139). In addition, point estimates suggested a positive association between logistic support and time spent in MVPA among boys (β = 5.71, 95% *CI* = 0.89 to 9.46) with the equivalent association close to the null among girls. The statistical evidence for this difference in association by gender was weak (*P* for Heterogeneity = 0.045).

The supplementary moderation analysis provided no evidence of a modeling support*nurturance interaction (β = −2.89, 95% *CI* = −6.51 to 0.73) or a logistic support*nurturance interaction (β = −0.51, 95% *CI* = −4.39 to 5.40).

## 4 Discussion

In this study, we found no evidence for an association between a nurturing parenting style and children's objectively assessed MVPA. This finding supports previous work that has found no association between authoritative parenting styles, of which nurturance is one dimension, and objectively-assessed MVPA among 5–10 year old children (Langer et al., 2014). The gender-stratified analysis suggested that the association between parent nurturance and MVPA may be inverse for girls. Whilst this result should be interpreted with caution as the sample sizes of boys and girls were modest, future research should investigate this potential difference further as it could indicate that parent nurturance (i.e., respecting a child's wishes and being responsive) may have different implications for the engagement in physical activity of boys and girls.

We found that parental nurturing was positively associated with the provision of modeling support (i.e., a parent being active themselves, role modeling physical activity), which supports a key pathway in the conceptual model of Patrick et al. (2013) and Sleddens et al. (2011). Langer et al. (2014) previously reported a positive association between an authoritative parenting style, which encompasses nurturance, and a parent support variable which included items reflective of both logistic and modeling support. These findings highlight the importance of analyzing the association between individual parenting practices and parenting styles.

We found evidence that parents' provision of modeling support was associated with children's MVPA. There was weaker evidence for an association between logistic support and MVPA, although the stratified analysis suggested that logistic support may be associated with boys' MVPA but not the MVPA of girls. While our findings suggest that parents play an important role in facilitating the physical activity of their young children, particularly through modeling, many adults/parents do not meet physical activity recommendations (Craig and Mindell, 2013) which may limit their opportunities to be an active role model. Paradoxically, having children is associated with lower levels of physical activity compared to adults with no children (Bellows-Riecken and Rhodes, 2008). While providing logistic support is possible for both active and less active parents, modeling of physical activity is only likely to come from active parents. As such, it is important that parenting interventions designed to increase children's physical activity also help parents identify activities that they value and enjoy to enhance their potential as a role model.

We identified a small mediation effect from parent nurturance through modelling support to children's MVPA. While these findings add some support for the proposed mediation model, the evidence is relatively weak compared to the direct effects of modeling on MVPA. For example, a one unit increase in modeling support was associated with a 2.4 min per day increase in child MVPA. The point estimates of the stratified analysis are suggestive of support for the mediation model via logistic support for boys only, although these results should be interpreted with caution due to low power to detect associations in the stratified analysis. If further prospective studies replicated these findings and there was support for causal effects from randomized controlled trials, then the magnitude of our findings suggest that an intervention would have to double the amount of parental modeling of those who provide little modeling support (i.e., those who score 1 or 2) in order to have an important impact on their child's MVPA. The nature of an intervention to achieve this change in parenting practices is something that should be explored in future studies; however the mediation analysis in the present study suggests that alternative antecedents to the provision of modeling support, beyond parenting styles, need to be identified.

Our supplementary analyses did not provide evidence to support the moderation hypothesis (i.e., that parenting style moderates the parenting practice-physical activity association) that has been identified in previous research (Hennessy et al., 2010, Langer et al., 2014). However these findings should be interpreted with a high level of caution due to the low power to detect interaction effects in this study, in addition to our limited measurement of parenting styles. Testing competing and/or complementary models such as those presented by Patrick et al. (2013) and Sleddens et al. (2011) using varied measures of parenting practices and robust measures of parenting styles in large samples is needed to extend this work further.

### 4.1 Limitations, strengths, and further work

To the best of our knowledge, this is the first study to test the mediation hypotheses of the recently proposed conceptual models of parenting influences on children's physical activity (Patrick et al., 2013, Sleddens et al., 2011). Other strengths of this study are its conceptual foundations, the large sample and the objective measurement of children's physical activity using accelerometers. Despite the strength of our objective measure of physical activity, the lack of consensus regarding which cut-points are most accurate for classifying MVPA among children and youth is a limitation of the literature, and inherently our study (Freedson et al., 2005, Kim et al., 2012). However, in following the current recommendations for researchers (Freedson et al., 2005, Kim et al., 2012), we chose the most appropriate MVPA cut-points based on our sample's age, and the methodology used in the original calibration study (Evenson et al., 2008). Some additional limitations should be noted. Firstly, on average, the children in our study met the UK physical activity guidelines and on average, parents reported that they provided positive support for their children's physical activity. As such, our findings are reflective of families that are supportive of activity and have children who are relatively more active. Parents included in the analysis were less deprived, and provided greater nurturing and logistic support than excluded parents. Further work is needed to develop strategies to ensure satisfactory adherence to data collection protocols, in particular accelerometer measures amongst children from families across the socioeconomic spectrum.

Secondly, our analysis of parenting dimensions was limited to nurturance only, because commensurate with previous work (Power, 2002), the measure of parental control showed low internal consistency. A potential reason for the low internal consistency is that the control scale adopts a dichotomous choice response format in which parents read two opposing statements and choose the one they agree with most. Parents may have found this response format confusing and therefore did not consistently select the response which reflected their view. Davison et al. (2013) highlight the challenges involved in measuring parenting styles, and recent advancements (Sleddens et al., 2014) have been made in developing more robust measures of general parenting which could be used in future work.

Thirdly, although we disaggregated parenting practices, we considered only logistic support and modeling: two positive practices from a myriad of parent behaviors (Trost et al., 2013). Within the measure of parent modeling, parent physical activity was assessed with a single item and results should be interpreted with caution. However, parent modeling is broader than parent behavior only and future research could combine objective measures of parent behavior, co-participation (i.e., concurrent measures of child and parent location and accelerometery) and subjective reports of parent attitudes towards physical activity. It would also be informative to examine associations between parenting styles and more controlling or restrictive parenting practices, as it is likely that parents may use such strategies when they are tired, busy, or facing resistance or challenging behavior of their children. Moreover, it would be valuable to consider a broader range of candidate antecedents to parenting practices than only parenting styles. There is a need to understand the personal, social, interpersonal, and environmental antecedents to how parents interact with their children to inform pragmatic intervention strategies involving parents.

Fourth, Sleddens et al. (2011) and Patrick et al. (2013) hypothesize a bidirectional relationship between parenting practices and diet/physical activity. Our mediation model was not able to examine this hypothesis and longitudinal studies are required to examine what is likely to be a dynamic and bidirectional process of parent and child interaction over time. As evidence suggests that mothers and fathers influence the psychosocial correlates of children's physical activity differently (Sebire et al., 2014), future research should study the dyadic or triadic influences of mothers' and fathers' parenting styles and practices on their children's physical activity. However, although our sample included a larger proportion of fathers (35%) than previous similar studies (Hennessy et al., 2010, Langer et al., 2014), it was not sufficiently large, neither did it include sufficient family triads to examine these associations within a comprehensive family subgroup breakdown. The majority of physical activity parenting literature is dominated by studies of maternal influence (Mitchell et al., 2012), and future work should include more fathers to gain a deeper understanding of family dynamics in relation to children's physical activity at different stages of development.

Finally, causality cannot be inferred from our findings due to the observational data, the potential biases in the mediator and the estimates of direct and indirect effects caused by measurement error in mediation models (le Cessie et al., 2012). For example, although we used previously validated measures (Davison et al., 2011, Power, 2002), parental nurturance and practices were measured by self–report, and scores are potentially similarly biased by socially desirable responding in which parents may report being more nurturing or providing greater activity support than they do. These sources of error may be correlated. Alternatively, unmeasured confounders (e.g., a child being unwell at the time of measurement) may influence both parents' perceptions of their activity support and children's activity.

## 5 Conclusions

This study demonstrated positive associations between logistic support and modeling parenting practices and the MVPA of 5–6 year old children. We have extended previous research by objectively measuring children's MVPA. A nurturing parenting style was associated with more frequent use of the measured parenting practices, and we found some evidence that parenting styles are associated with children's MVPA through parenting practices. By studying the mechanisms which are involved in physical activity parenting, our work tentatively suggests that parenting interventions aimed at increasing young children's MVPA would do well to focus on fostering both the *what* (i.e., positive parenting practices) and the *how* (i.e., parenting styles). At the same time, future research should seek to identify a broader range of factors that are associated with parents' use of positive physical activity parenting practices.

## Figures and Tables

**Table 1:** Descriptive characteristics of the included sample (*N* = 770).

Variable	Mean (*SD*)	95% *CI*
Child age (years)	6.00 (0.43)	5.97, 6.03
Child BMI (SDS)	0.21 (0.92)	0.14, 0.27
Area IMD (mean score)	13.28 (10.88)	12.47, 14.00
Child MVPA (minutes/day)	67.16 (19.49)	65.82, 68.57
Parent nurturance (total score; maximum score 36)	31.92 (3.87)	31.64, 32.19
Parent nurturance (maximum score 6)	5.32 (0.64)	5.27, 5.36
Parent logistic support (maximum score 4)	3.12 (0.67)	3.07, 3.17
Parent modeling support (maximum score 4)	3.42 (0.53)	3.39, 3.47

Note. Descriptive characteristics are presented as Mean (Standard Deviation [SD]) unless otherwise stated.

Abbr. BMI = Body mass index. *CI* = Confidence interval. IMD = Index of multiple deprivation. MVPA = Moderate-to-vigorous physical activity. SD = Standard Deviation. SDS = Standard deviation score.

**Table 2:** Linear regression analyses showing direct and indirect (mediated) associations between parenting style, practices and children's moderate-to-vigorous physical activity.

	Unstandardized β (Robust SE^a^)	95% *CI*	*p*
Step 1.			
Parental nurturance → child MVPA	−1.43 (1.22)	−3.89, 1.03	0.251
	*R*^*2*^ = 0.079		<0.001
Step 2.			
Parental nurturance → modeling support	0.11 (0.05)	0.02, 0.21	0.017
	*R*^*2*^ = 0.029		0.006
Parental nurturance → logistic support	0.14 (0.03)	0.07, 0.21	<0.001
	*R*^*2*^ = 0.041		0.001
Step 3.			
Modeling support → child MVPA^b^	2.41 (1.09)	0.23, 4.60	0.031
Logistic support → child MVPA^b^	2.41 (1.41)	−0.42, 5.25	0.09
	*R*^*2*^ = 0.032		<0.001
Indirect (Mediation) effects:			
Indirect effect 1 (nurturance → modeling → child MVPA)	0.27 (0.16)	0.04, 0.70^c^	
Indirect effect 2 (nurturance → logistic → child MVPA)	0.34 (0.22)	−0.02, 0.83^c^	
Total indirect effect	0.61		

Note. All models are adjusted for child gender, parent's gender, index of multiple deprivation, and child BMI z-score.

Abbr. CI = confidence interval. SE = standard error. MVPA = moderate to vigorous physical activity.

a Robust SE is adjusted for clustering.

b Also adjusted for parental nurturance and the other mediator.

c Bias-corrected 95% *CI*.
