# Supplementary material for: Examining a conceptual model of parental nurturance, parenting practices and physical activity among 5–6 year olds
Source: Soc Sci Med. 2016 Jan;148:18–24. doi: 10.1016/j.socscimed.2015.11.022 (PMC4714610; doi:10.1016/j.socscimed.2015.11.022)
Supplement: Supplementary file 1 [file mmc1.docx]

**Supplemental Table 1.** **Linear regression analyses, stratified by gender, showing direct and indirect (mediated) associations between parenting style, practices and children’s moderate-to-vigorous physical activity.**

|  | **Boys** | | **Girls** | |  |
| --- | --- | --- | --- | --- | --- |
|  | **Unstandardized β**  **(robust SE ^a^)** | **95% *CI*** | **Unstandardized β**  **(robust SE ^a^)** | **95% *CI*** | ***P* for Heterogeneity** |
| Step 1. |  |  |  |  |  |
| Parental nurturance → child MVPA | 0.15 (1.86) | [-3.58, 3.89] | -2.90 (1.47) | [-5.84, 0.04] | 0.139 |
|  | ***R^2^* = 0.044** | ***p*=0.001** | ***R^2^* = 0.02** | ***p*=0.186** |  |
| Step 2. |  |  |  |  |  |
| Parental nurturance → modelling support | 0.13 (0.63) | [0.01, 0.26] | 0.09 (0.61) | [-0.03, 0.22] | 0.784 |
|  | ***R^2^* = 0.043** | ***p*<0.001** | ***R^2^* = 0.020;** | ***p*<0.284** |  |
| Parental nurturance → logistic support | 0.13 (0.05) | [0.04, 0.23] | 0.14 (0.05) | [0.04, 0.25] | 0.739 |
|  | ***R^2^* = 0.030** | ***p*<0.018** | ***R^2^* = 0.050** | ***p*<0.003** |  |
| Step 3. |  |  |  |  |  |
| Modelling support → child MVPA^b^ | 1.29 (1.62) | [-1.96, 4.55] | 3.75 (1.25) | [1.24, 6.25] | 0.747 |
| Logistic support → child MVPA^b^ | 5.17 (2.13) | [0.89, 9.46] | -0.40 (1.58) | [-3.58, 2.76] | 0.045 |
|  | ***R^2^* = 0.066** | ***p*=0.001** | ***R^2^* = 0.04** | ***p*<0.075** |  |
| ***Indirect (Mediation) effects****:* |  |  |  |  |  |
| Indirect effect 1  (nurturance → modelling → child MVPA) | 0.17 (0.25) | [-0.18, 0.86] ^c^ | 0.35 (0.25) | [-0.10, 0.94] ^c^ |  |
| Indirect effect 2  (nurturance → logistic → child MVPA) | 0.69 (0.37) | [0.14, 1.67] ^c^ | -0.06 (0.26) | [-0.63, 0.46] ^c^ |  |
| Total indirect effect | 0.86 |  | 0.29 |  |  |

***Note.*** All models are adjusted for child gender, parent’s gender, index of multiple deprivation, and child BMI *z*-score. ***Abbr.*** *CI*= confidence interval. SE = standard error. MVPA = moderate to vigorous physical activity. ^a^ Robust SE is adjusted for clustering. ^b^ Also adjusted for parental nurturance and the other mediator. ^c^ Bias-corrected 95% *CI*.
